# Clonal hematopoiesis in AML long‐term survivors: Risk factors and clinical consequences

**DOI:** 10.1002/hem3.70183

**Published:** 2025-07-24

**Authors:** Simon M. Krauß, Eva Telzerow, Daniel Richter, Anna S. Moret, Maja Rothenberg‐Thurley, Cristina Sauerland, Anne Weigert, Alessia Fraccaroli, Johanna Tischer, Frank Ziemann, Katharina S. Götze, Wolfgang E. Berdel, Bernhard Wörmann, Utz Krug, Jan Braess, Pia Heussner, Wolfgang Enard, Wolfgang Hiddemann, Karsten Spiekermann, Dennis Görlich, Uwe Platzbecker, Klaus H. Metzeler

**Affiliations:** ^1^ Department of Hematology, Cell Therapy, Hemostaseology and Infectiology University of Leipzig Medical Center Leipzig Germany; ^2^ Department of Medicine III, University Hospital Ludwig‐Maximilians‐Universität München Munich Germany; ^3^ Anthropology and Human Genomics, Faculty of Biology Ludwig‐Maximilians‐Universität München Munich Germany; ^4^ Institute of Biostatistics and Clinical Research University of Münster Münster Germany; ^5^ Department of Medicine III, TUM University Hospital Technical University of Munich Munich Germany; ^6^ Department of Medicine, Hematology and Oncology University of Münster Münster Germany; ^7^ Deutsche Gesellschaft für Hämatologie und Medizinische Onkologie Berlin Germany; ^8^ DKMS Collection Center Cologne Germany; ^9^ Department of Oncology and Hematology Hospital Barmherzige Brüder Regensburg Germany; ^10^ Psycho‐Oncology Hospital Garmisch‐Partenkirchen Garmisch‐Partenkirchen Germany; ^11^ German Cancer Consortium (DKTK), Partner Site Munich a Partnership Between DKFZ and LMU University Hospital Munich Germany; ^12^ Partner Site Munich Bavarian Cancer Research Center (BZKF) Munich Germany; ^13^ University Hospital Carl Gustav Carus Technische Universität Dresden Dresden Germany

## Abstract

Clonal hematopoiesis (CH) is common in the general population and associated with various health risks, but its prevalence and clinical implications in acute myeloid leukemia (AML) long‐term survivors (LTS; ≥5‐year survival) are unknown. We analyzed CH in 373 AML‐LTS with a median 11.6‐year follow‐up from diagnosis using a sensitive targeted sequencing assay based on single‐molecule molecular inversion probes. CH variants were detected in 61.9% of survivors, with 26% having small‐clone CH (SC‐CH, variant allele frequency (VAF) < 2%) and 35.9% CH of indeterminate potential (≥2% VAF). CH was more prevalent in survivors treated with chemotherapy only (75.7%) compared to those who received allogeneic stem cell transplantation (alloSCT, 54.0%) and to age group‐matched healthy controls. In chemotherapy‐treated survivors, CH prevalence increased with age, whereas in alloSCT recipients, it most closely associated with hematopoietic age (i.e., the sum of donor age and time since transplantation). The variant spectrum also differed by treatment, with *TP53* and *PPM1D* variants being more common in the chemotherapy group. CH variants ≥10% VAF associated with increased risks of diabetes in alloSCT recipients and secondary neoplasms in chemotherapy‐treated survivors. This study provides insights into the high prevalence and potential clinical relevance of CH in AML‐LTS.

## Graphical abstract

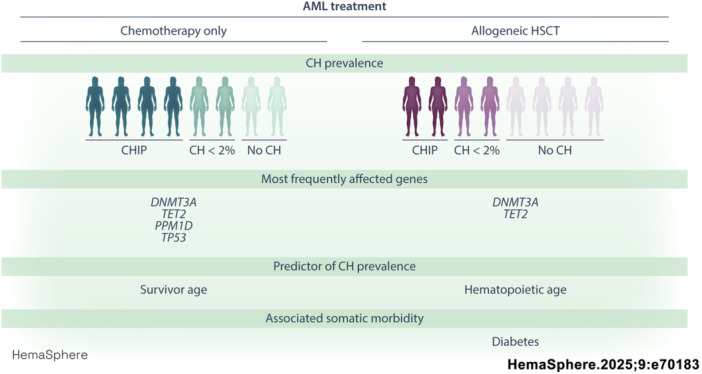

## INTRODUCTION

Clonal hematopoiesis (CH) is a common aging‐related phenomenon in the general population. It is defined as the outgrowth of a clonal population of blood cells, driven by somatically acquired gene mutations, including single‐nucleotide variants and short insertions/deletions, in the absence of overt hematopoietic malignancies. CH prevalence and clone size increase with age, and recent studies suggest that CH is nearly ubiquitous in individuals aged 70 or older.^1^, ^2^, ^3^, ^4^, ^5^ CH mutations most frequently affect epigenetic regulators (including *DNMT3A*, *TET2*, and *ASXL1*, often referred to as the “DTA” genes) and, less commonly, RNA splicing factors (e.g., *SF3B1* and *SRSF2*) and other genes.^5^ In patients treated with chemotherapy or radiation therapy, a distinct type of CH mutations is found in genes involved in DNA damage response pathways (*PPM1D*, *TP53*, and *CHEK2*).^6^, ^7^ Studies in pediatric cancer survivors have shown that prevalence of CH is increased compared to age‐matched controls, persists for more than 5 years, and have linked this higher frequency to treatment with alkylating agents.^8^, ^9^ CH with a variant allele frequency (VAF) of ≥2% in the absence of cytopenia has been termed “clonal hematopoiesis of indeterminate potential” (CHIP), and most studies investigating the clinical effects of CH have focused on CHIP mutations. CHIP and clonal cytopenia of undetermined significance are premalignant states carrying a variable, but elevated risk of progression to overt hematologic malignancies including myelodysplastic neoplasia (MDS) and acute myeloid leukemia (AML).^5^ Importantly, CH is also associated with an increased risk of non‐hematologic diseases, including cardiovascular disease and various inflammatory disorders.^10^, ^11^, ^12^, ^13^ Overall, CHIP has been linked to an increased overall mortality.^2^, ^14^

In AML patients, the emergence of preleukemic clones carrying CH mutations often predates AML diagnosis by many years. CH mutations can persist during and after induction chemotherapy in patients achieving remission^15^, ^16^, ^17^; however, little is known regarding the presence and clinical significance of CH in AML survivors. Over the last decades, 5‐year (y) survival rates for AML patients < 60 y have increased substantially, now ranging from 30% to 50% depending on age.^18^, ^19^ As a result, an increasing number of AML patients are expected to become long‐term survivors (LTS), underscoring the need for research on their long‐term health‐related challenges.^20^ Given its association with various hematologic and non‐hematologic diseases, particularly cardiovascular and inflammatory disorders, and its potential persistence during AML remission, a better understanding of CH in AML‐LTS is needed. We therefore conducted a retrospective, multicentric study on a large cohort of AML‐LTS ≥ 5 y after initial diagnosis to systematically assess the prevalence of CH and its association with somatic late and long‐term morbidity. Using a highly sensitive targeted sequencing assay, which utilizes single‐molecule molecular inversion probes (smMIPs) and computational error correction via unique molecular identifiers (UMIs), we aimed to detect even low‐frequency clones to ensure a comprehensive analysis of CH in AML survivors. Our study aimed to identify determinants of CH and its potential impact on long‐term health outcomes of AML survivors.

## METHODS

### Study population

Participants were recruited into a cross‐sectional study of quality of life and late and long‐term effects in AML‐LTS. Key inclusion criteria were age ≥ 18 y, an initial diagnosis of AML ≥ 5 y before participation in the survivorship study, and no current AML relapse requiring treatment. All study participants were formerly enrolled in clinical trials of the German AML Cooperative Group (AMLCG‐1999,^21^ clinicaltrials.gov identifier NCT00266136; AMLCG‐2004,^22^ European Leukemia Trial Registry identifier LN_AMLINT_2004_230; and AMLCG‐2008,^23^ NCT01382147), or the AMLCG patient registry (DRKS identifier DRKS00020816). Of 427 former patients enrolled in the survivorship study, 378 agreed to voluntarily provide a peripheral blood sample. Information on somatic health status was collected from patients and their primary care physicians using questionnaires aligned with items from the German population‐based DEGS1 health survey,^24^ complemented by copies of recent medical reports when available. This study was approved by the responsible ethics committee at the University of Munich (reference no. 17‐444) and registered in the German Clinical Trials Registry (DRKS, www.drks.de, identifier DRKS00023991). All participants provided written informed consent. Control samples from 137 individuals without known hematologic neoplasia were obtained from the Laboratory of Leukemia Diagnostics, University of Munich.

### Analysis of CH via NGS

Genetic variants associated with CH were identified from genomic DNA from peripheral blood mononuclear cells, using a targeted sequencing assay based on smMIPs.^25^ SmMIPs were designed to cover 82 genomic loci in 24 CH‐related genes (genomic target size, approx. 16 kb; Supporting Information S2: Tables 1 and 2). Details on smMIP assay design, library preparation, sequencing, quality control, data processing, and variant calling, filtering, and annotation are provided in the supplementary material. The median sequencing depth was 8970x before and 900x after UMI‐deduplication, with 95% of the target regions covered ≥960x before and ≥124x after UMI‐deduplication. We grouped survivors with detectable variants into two groups: those with a maximum VAF of ≥2% were classified as having CHIP, whereas those with a maximum VAF < 2% were designated as small‐clone CH (SC‐CH). We use the term “CHIP” despite the survivors' history of hematologic neoplasm, as they were in long‐term remission at study inclusion.

### Statistical analyses

Survivors without CH, with SC‐CH, or with CHIP were compared using the Kruskal–Wallis rank sum test for continuous variables and Pearson's chi‐squared test or Fisher's exact test for categorical variables. When analyzing age, age at study inclusion was used for survivors, and age at allogeneic transplantation was used for donors, if not specified otherwise. In transplanted survivors, “hematopoietic age”^26^ was calculated as the sum of donor age and time elapsed since transplantation. Ordinal logistic regression models with a logit link function were used to identify factors associated with CH status. The association between CH status and somatic morbidity was assessed using univariable and multivariable logistic regression models. We report 95% confidence intervals (CIs) for computed odds ratios of (ordinal) logistic regression models, and the Akaike information criterion (AIC) was used to assess model fit. Kaplan–Meier curves are presented as 1 minus event‐free probability to indicate the cumulative incidence of secondary neoplasms. Cox proportional hazards regression with hazard ratios and pointwise 95% CI were applied to study the incidence of secondary neoplasms, and log‐rank tests were used to compare time‐to‐event curves. Adjustment for multiple comparisons was performed when appropriate using the Benjamini–Hochberg method to control the false‐discovery rate (*q*‐value), and variables with a P‐value < 0.05 and an associated *q*‐value < 0.1 were considered statistically significant. All statistical analyses were conducted using the R statistical computing language (version 3.6.0/4.4.1).^27^

## RESULTS

### CH is common in AML‐LTS

CH analysis was successful in 373 of 378 AML‐LTS with available peripheral blood samples (Table 1), with a median age of 49 y (range, 17–80 y) at initial AML diagnosis and 61 y (range, 28–93 y) at survivorship study enrollment, corresponding to a median follow‐up of 11.6 y (range, 5.3–19.8 y). All patients had received cytarabine‐ and anthracycline/anthracenedione‐based induction chemotherapy, and high‐dose cytarabine was used during induction in 88%. Of note, 37% of survivors underwent consolidation chemotherapy and/or an autologous stem cell transplant (performed in 5.9%), but never received an allogeneic transplant (subsequently referred to as the ‘*chemo*’ subgroup). Sixty‐three percent had received an allogeneic hematopoietic stem cell transplantation, either in first remission or for relapsed/refractory disease (the ‘*alloSCT*’ subgroup). *Chemo* survivors had received significantly more cycles of consolidation (P < 0.001) and maintenance (P < 0.001) therapy, but we observed no difference in the number of induction therapy cycles (Supporting Information S1: Table 4). Chemotherapy treatment regimen details, number of treatment lines, and information about allogeneic HSCT donor matching status are shown in the supplement (Supporting Information S1: Tables 4–6).

**Table 1:** Survivor characteristics by clonal hematopoiesis (CH) status.

Variable	Overall	No CH	SC‐CH	CHIP	P
Survivor number	*N* = 373^a^	*n* = 142^a^	*n* = 97^a^	*n* = 134^a^	
Age at initial diagnosis (y)	49 (17, 80)	49 (17, 76)	49 (18, 74)	51 (18, 80)	0.3^b^
Age at CH analysis (y)	61 (28, 93)	59 (28, 91)	61 (28, 87)	62 (32, 93)	**0.038** b
Years since AML diagnosis	11.6 (5.3, 19.8)	8.2 (5.3, 18.6)	12.2 (5.6, 19.8)	12.3 (5.5, 18.4)	**<0.001** b
Sex					**0.027** c
Female	216	87 (40%)	45 (21%)	84 (39%)	
Male	157	55 (35%)	52 (33%)	50 (32%)	
Smoking	61	16 (26%)	20 (33%)	25 (41%)	0.2^c^
Unknown	61	28	13	20	
Treatment					**<0.001** c
chemo	136	33 (24%)	30 (22%)	73 (54%)	
alloSCT	237	109 (46%)	67 (28%)	61 (26%)	
AML type					**0.008** c
De novo AML	313	109 (35%)	83 (27%)	121 (39%)	
sAML/tAML	60	33 (55%)	14 (23%)	13 (22%)	
Cytogenetic risk group					0.6^c^
Favorable	51	23 (45%)	10 (20%)	18 (35%)	
Intermediate	266	95 (36%)	72 (27%)	99 (37%)	
Adverse	56	24 (43%)	15 (27%)	17 (30%)	
Leukocyte counts at initial diagnosis (×10^9^/L)	10.2 (0.6, 391.2)	8.6 (0.8, 284.0)	8.7 (0.6, 244.4)	13.1 (0.6, 391.2)	0.8^b^
Unknown	12	2	4	6	
AMLCG trial					**<0.001** c
1999	215	61 (28%)	64 (30%)	90 (42%)	
2004	24	8 (33%)	6 (25%)	10 (42%)	
2008	67	33 (49%)	14 (21%)	20 (30%)	
Register	67	40 (60%)	13 (19%)	14 (21%)	

*Note:* Statistically significant values (P < 0.05) are indicated in bold.

Abbreviations: alloSCT, allogeneic stem cell transplantation; AML, acute myeloid leukemia; AMLCG, AML Cooperative Group; CHIP, clonal hematopoiesis of indeterminate potential; sAML, secondary acute myeloid leukemia; SC‐CH, small‐clone clonal hematopoiesis; tAML, therapy‐related acute myeloid leukemia.

^a^ Median (range); *n* (row %).

^b^ Kruskal–Wallis rank sum test.

^c^ Pearson's chi‐squared test.

**Figure 1:**
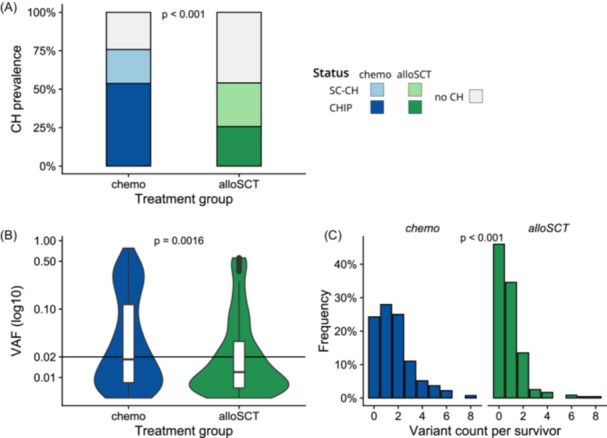
**Comparison of clonal hematopoiesis (CH) prevalence, variant allele frequency (VAF), and variant count between treatment groups. (A)** Stacked bar plot comparing prevalence of CH of indeterminate potential (CHIP) and small‐clone CH (SC‐CH) in *chemo* and allogeneic stem cell transplantation (*alloSCT*) groups. **(B)** Violin‐boxplot comparing VAF between *chemo* and *alloSCT* groups. **(C)** Histogram comparing variant count per survivor between *chemo* and *alloSCT* groups.

In our cohort, 231 AML survivors (61.9%) carried at least one CH‐related variant, including 97 with SC‐CH (<2% VAF; 26%) and 134 with CHIP (≥2% VAF; 35.9%). In total, we detected 437 variants (median VAF, 1.5%; range, 0.5%–78.0%; Supporting Information S2: Table 3), with 120 survivors carrying one, 66 carrying two, and 45 carrying three or more variants (Supporting Information S1: Figure 1). On average, survivors affected by CH carried 1.9 variants.

We observed an association of CH prevalence with age, as survivors with CH were significantly older at the time of blood sample collection than those with no detectable variants (P = 0.038, Table 1). Prevalence of CH also associated with longer time since AML diagnosis (P < 0.001). Conversely, age at the time of initial leukemia diagnosis showed no association with CH status (Table 1). CHIP was slightly more common in female than in male survivors.

**Figure 2:**
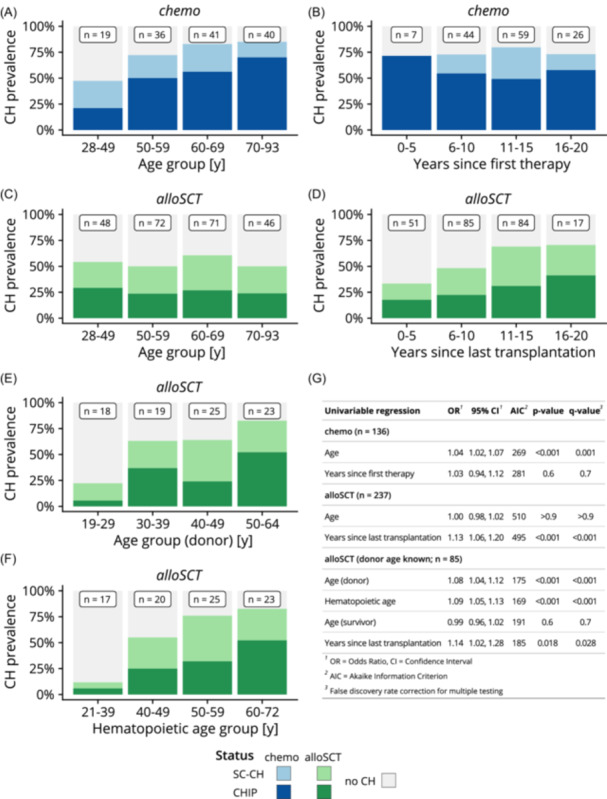
**Overview of time factors associated with clonal hematopoiesis (CH) prevalence. (A, B)** Stacked barplots displaying CH prevalence in *chemo* survivors in dependence of either age group or years since first chemotherapy. **(C, D)** Stacked barplots displaying CH prevalence in survivors treated with allogeneic stem cell transplantation (*alloSCT*) in dependence of either age group or years since last allogeneic transplantation. **(E, F)** Stacked barplots displaying CH prevalence in a subset of *alloSCT* survivors with known donor age, either in dependence of donor age at transplantation or hematopoietic age, that is, the sum of donor age and time since transplantation. **(G)** Univariable ordinal logistic regression analysis of the same factors as in **(A)**–**(F)**. Age was treated as a continuous variable, and odds ratio (OR) is shown per year. CHIP, clonal hematopoiesis of indeterminate potential; SC‐CH, small‐clone clonal hematopoiesis.

Hematologic parameters generally were similar in survivors with and without CH, with no significant differences in leukocyte, neutrophil, or platelet counts or mean corpuscular volume. Hemoglobin levels differed between the subgroups of survivors without CH, with SC‐CH, or with CHIP (P = 0.002), yet the absolute difference in median hemoglobin concentration was small and directionally discordant between survivors with SC‐CH and CHIP, and the clinical relevance of this finding is questionable (Supporting Information S1: Figure 2).

### Factors affecting prevalence and spectrum of CH variants in AML survivors

We observed differences in the prevalence and variant spectrum of CH according to the modality of antileukemic treatment that survivors had received. In survivors of the *chemo* group, CH was significantly more common than in *alloSCT* survivors (75.7% vs. 54.0%; P < 0.001). Although the number of survivors with SC‐CH was comparable between both groups (22.1% vs. 28.3%; P = 0.22), the number of survivors with CHIP was more than two times higher in the *chemo* group than in the *alloSCT* group (53.7% vs. 25.7%; P < 0.001; Figure 1A). Concordantly, the median VAF was significantly higher in *chemo* survivors than in *alloSCT* survivors (1.8% vs. 1.2%; P = 0.0016; Figure 1B) and on average, *chemo* survivors also carried a higher number of variants (2.2 vs. 1.6 average variants within survivors affected by CH; P < 0.001; Figure 1C). Due to this clear influence of leukemia treatment modality on CH status, we stratified survivors into these two treatment groups for all subsequent analyses. Baseline characteristics of *chemo* and *alloSCT* survivors are compared in Supporting Information S1: Table 7.

In the *chemo* group, we observed an increase in CH prevalence with higher age at the time of CH analysis (<50 y, 50–59 y, 60–69 y, and ≥70 y), from 47.4% in the youngest to 85.0% in the oldest age group. This was mostly driven by an increase in CHIP prevalence, which rose from 21.1% to 70.0%, while the prevalence of SC‐CH decreased from 26.3% to 15.0% (Figure 2A). Conversely, the prevalence of CH in the *alloSCT* group did not increase with age at the time of analysis (Figure 2C). Using ordinal logistic regression, we confirmed a significant association between age as a continuous variable and CH status (no CH vs. SC‐CH vs. CHIP) for *chemo* survivors (odds ratio [OR], 1.04 per age year; P < 0.001). In contrast, for *alloSCT* survivors, there was no significant association between age and CH category (OR, 1.0; P > 0.90; Figure 2G).

**Figure 3:**
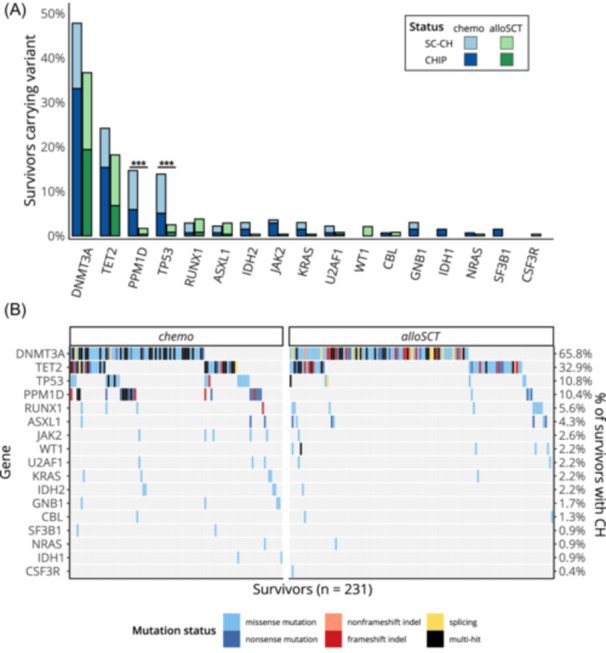
**Distribution of clonal hematopoiesis (CH)‐associated genetic variants stratified by treatment modality. (A)** Proportion of survivors of their respective treatment group carrying CH variants in specific genes. **(B)** Oncoplot depicting variants across all 231 affected survivors, stratified by treatment modality. Each column represents one survivor, while each row represents a specific gene, with coloring indicating the variant type. Percentages on the right axis denote the frequency of gene variants within the group of survivors affected by CH. alloSCT, allogeneic stem cell transplantation; CHIP, clonal hematopoiesis of indeterminate potential; SC‐CH, small‐clone clonal hematopoiesis.

When compared to a cohort of healthy controls studied with the same assay, *chemo* survivors exhibited a higher CH prevalence across all age groups. Multivariable ordinal logistic regression, adjusted for age, confirmed that *chemo* survivors were more likely to be in a more advanced CH status category than controls (OR 3.53, P < 0.001; Supporting Information S1: Figure 3).

Next, we assessed whether CH status was associated with time since therapy, defined as time since first chemotherapy in the *chemo* group or time elapsed since last allogeneic transplantation in the *alloSCT* group. Although overall prevalence of CH did not increase with time since therapy in the *chemo* group (Figure 2B), prevalence of CH in *alloSCT* recipients increased with time since transplant, from 33.3% (≤5 y from transplantation) to 70.6% (>15 y; Figure 2D), with the increase driven by both SC‐CH (rising from 15.7% to 29.4%) and CHIP (rising from 17.6% to 41.2%). Again, these results were reflected in the corresponding ordinal logistic regression models. Although the model for the *chemo* group did not show an association between time since first therapy and CH prevalence (OR, 1.03; P = 0.60), the model for the *alloSCT* group indicated a 13% increased risk of more advanced CH status for each year since transplantation (OR, 1.13; P < 0.001; Figure 2G). As hematopoiesis in *alloSCT* AML survivors is derived from donor stem cells, we reasoned that the age of the stem cell donor could be a predictor of the *alloSCT* survivors' CH status. We were able to determine donor age at the time of transplantation for 85 of 237 *alloSCT* survivors, and observed a clear association of donor age group with overall CH prevalence in survivors, rising from 22.2% for donors aged < 29 y to 82.6% for donors aged ≥ 50 y. Although SC‐CH prevalence increased from 16.6% to 30.4%, CHIP prevalence increased from 5.6% to 52.2% between these donor age groups (Figure 2E). An ordinal logistic regression model confirmed these findings (OR, 1.08; P < 0.001; Figure 2G). We then calculated the “hematopoietic age” of the survivors' hematopoietic stem cell compartment at the time of CH analysis as defined by Oshima et al.,^26^ that is, the sum of donor age at transplantation and time elapsed since transplantation. Overall CH prevalence increased with hematopoietic age, from 11.8% for hematopoietic age < 40 y to 82.6% for hematopoietic age ≥ 60 y. The prevalence of SC‐CH and CHIP increased from 5.9% to 30.4% and from 5.9% to 52.2%, respectively (Figure 2F). Ordinal logistic regression again confirmed these results (OR, 1.09; P < 0.001; Figure 2G). Among the 85 survivors with known donor age, hematopoietic age at the time of CH analysis emerged as the variable most closely associated with CH status, based on the lowest AIC indicating the best model fit (Figure 2G).

**Figure 4:**
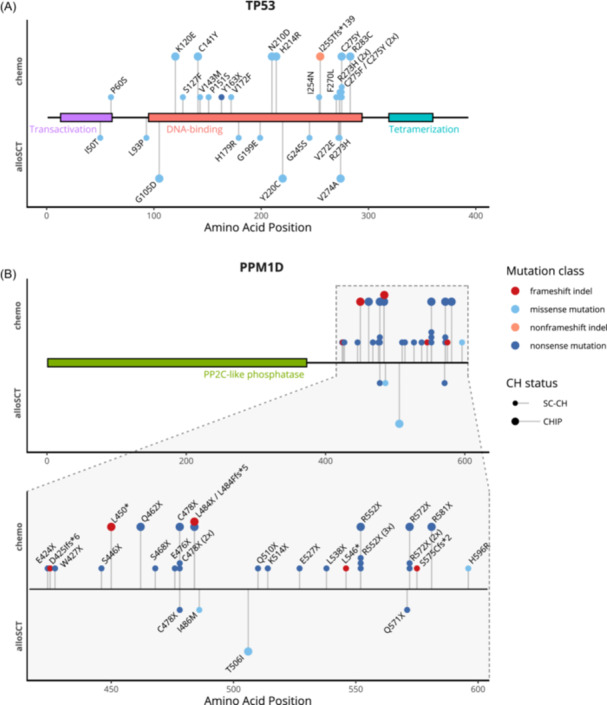
**Lollipop plot displaying distribution and classification of *TP53* and *PPM1D* variants.** Protein domains are indicated by colored regions. Amino acid changes are shown based on the canonical reference transcript. Variants are colored and classified by type and stratified by treatment modality. Variant size (i.e., clonal hematopoiesis [CH] status) is indicated by lollipop size and stem length. Multiple variants at the same position are displayed as stacked lollipops. **(A)** *TP53* (reference transcript: NM_000546; domains: InterPro P04637). One splice site mutation (c.375+2T>G) is not shown due to missing amino acid annotation, and one frameshift deletion (NM_001276696:exon10:c.897delA:p.E300Kfs*11) is not shown due to missing annotation to the canonical reference transcript. **(B)** *PPM1D* (reference transcript: NM_003620; domains: InterPro O15297). alloSCT, allogeneic stem cell transplantation; CHIP, clonal hematopoiesis of indeterminate potential; SC‐CH, small‐clone clonal hematopoiesis.

Our data indicated that related alloSCT donors were significantly older than unrelated donors (median, 47 vs. 37 y; P = 0.001). Survivors with related donors were more likely to carry CHIP variants compared to those with unrelated donors (P = 0.018; Supporting Information S1: Figure 4A,B). However, multivariable ordinal logistic regression models adjusted for relation status showed that hematopoietic age was a better predictor of CH status than donor type (Supporting Information S1: Figure 4C,D).

In summary, our results indicate that CH prevalence is most closely associated with patient age in *chemo* survivors, and with hematopoietic age in *alloSCT* survivors.

**Figure 5:**
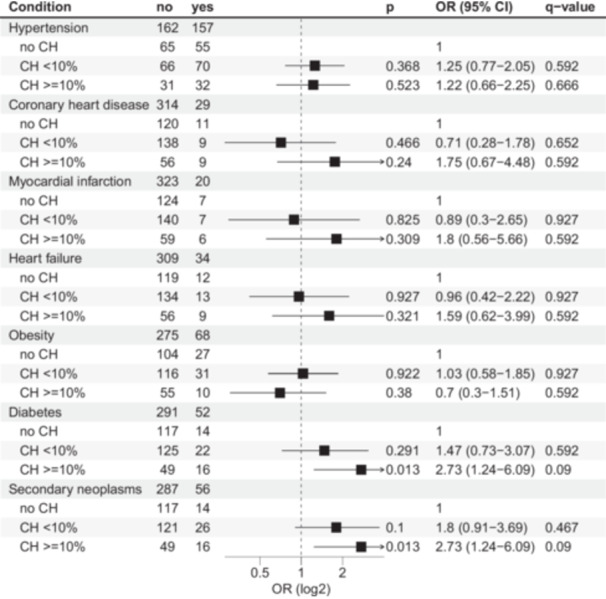
**Association between clonal hematopoiesis (CH) and somatic comorbidities presented as forest plot.** Odds ratio (OR), CI, and P‐values were calculated via univariable logistic regression. No/yes columns display the number of survivors affected by each comorbidity.

**Figure 6:**
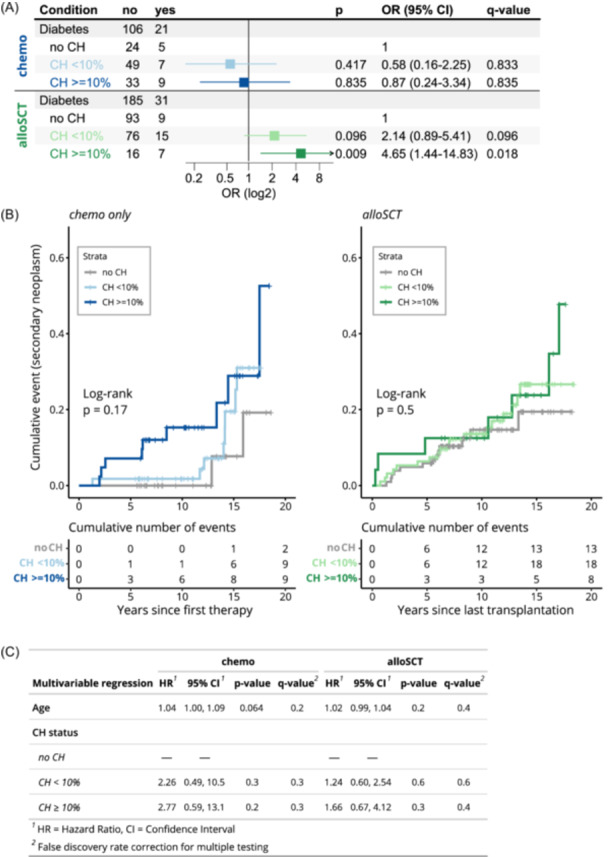
**Association of clonal hematopoiesis (CH) with diabetes and secondary neoplasms stratified by treatment modality. (A)** Forest plot displaying the association of diabetes with CH status stratified by treatment modality. Odds ratio (OR), CI, and P‐values were calculated via multivariable logistic regression adjusting for survivor age. **(B)** Cumulative event curves depicting the cumulative incidence of secondary neoplasms in survivors stratified by CH status and treatment modality over 20 years following the first chemotherapy (*chemo* group) or the last allogeneic transplantation (*alloSCT* group). Tick marks represent censored observations. **(C)** Results of multivariable Cox proportional hazards analysis for the risk of developing secondary neoplasms based on CH status and adjusted for age, stratified by treatment modality.

Univariable linear regression analysis using the largest VAF per survivor instead of categorical SC‐CH/CHIP status as the dependent variable produced comparable trends, with survivor age being the best predictor of maximum VAF in *chemo* survivors, but after correction for multiple testing, only hematopoietic age stayed significant as the best predictor of max VAF in *alloSCT* survivors (beta, 0.002; P = 0.005; Supporting Information S1: Figure 5). Similarly, survivor age (beta, 0.028; P < 0.001) and hematopoietic age (beta, 0.051; P < 0.001) emerged as the best predictors of variant count per survivor in *chemo* and *alloSCT* survivors, respectively (Supporting Information S1: Figure 6).

### Variant spectrum is influenced by treatment

The most commonly mutated gene in our cohort was *DNMT3A*, followed by *TET2*, *TP53*, and *PPM1D* (Figure 3A). The variant spectrum differed according to treatment group, as both *TP53* and *PPM1D* were more commonly found mutated in the *chemo* group than in the *alloSCT* group (multiple testing‐adjusted P < 0.001 for both). Most variants in both genes were SC‐CH variants. Further analysis showed that *PPM1D* and *TP53* variants could occur simultaneously in the same patient, as singular variants, or together with variants in *DNMT3A* and *TET2* (Figure 3B). The *alloSCT* group exhibited a higher proportion of single‐hit *DNMT3A* and *TET2* variants, mimicking the well‐established mutation pattern of age‐dependent CH. Codon R882 alterations accounted for 21% (21/98) of *DNMT3A* variants in *chemo* survivors but for only 11% (12/108) in *alloSCT* survivors (P = 0.057). *TP53* variants were mostly located in its DNA‐binding domain, whereas *PPM1D* variants were exclusively located downstream of its PP2C‐like phosphatase domain (Figure 4). Of note, *PPM1D* variants in *chemo* survivors were exclusively truncating (nonsense, frameshift indels, or multihit) variants, whereas two of four alloSCT survivors with *PPM1D* mutations carried missense variants.

### Association of CH with somatic morbidity in AML‐LTS

Next, we aimed to analyze associations of CH with somatic health status in 343 survivors with available data. We did not detect an association between smoking and CH status in the entire cohort (Table 1), nor in the subgroup of *chemo* survivors (data not shown). An analysis of associations between CH status and hypertension, coronary heart disease, myocardial infarction, obesity, diabetes, and secondary neoplasms via univariable logistic regression did not reveal any significant results after correction for multiple testing (Supporting Information S1: Figure 7). As previous studies^28^ suggest that larger CH mutations are more strongly associated with adverse clinical outcomes, we repeated this analysis with survivors grouped by CH status as no CH, CH < 10% VAF, and CH ≥ 10% VAF (Figure 5). We found significant associations of CH ≥ 10% with diabetes (OR, 2.73; P = 0.013) and secondary neoplasms (OR, 2.73; P = 0.013). As we had already observed an influence of treatment modality on CH status, we further investigated these somatic diseases in the treatment subgroups. Diabetes was not associated with CH status in *chemo* survivors, while it was significantly more prevalent in *alloSCT* survivors with CH ≥ 10% (OR, 4.65; P = 0.009; Figure 6A). In contrast, cumulative incidence analysis did not confirm the link between secondary malignancies and CH variants ≥ 10% VAF in chemo survivors (Figure 6B), especially after adjusting for survivor age in Cox proportional hazards regression (Figure 6C).

## DISCUSSION

### CH overall and in cancer

To the best of our knowledge, there are no published data on the prevalence of CH in cohorts of AML‐LTS (i.e., ≥5 y) that are not restricted to specific treatment modalities, such as allogeneic transplantation. Shorter term studies in AML patients who recently underwent induction chemotherapy have shown that CH clones, often related to the leukemia clone, frequently persist during hematologic remission and are associated with higher incidence of relapse and shorter overall survival.^16^, ^17^ Patients with *TET2* CH also showed prolonged neutropenia.^16^ However, data on the clinical relevance of CH in AML‐LTS are scarce.

In our cohort, we identified a high prevalence of CH in AML‐LTS. Treatment modality significantly influenced CH prevalence, as survivors who had been treated with chemotherapy without allogeneic transplantation exhibited a higher prevalence than those who additionally received alloSCT. Since detection of somatic mutations is highly dependent on the applied sequencing technique and analytical approach, its sensitivity and the spectrum of covered genes, comparison of our results with other cohorts is difficult. Notably, *chemo* survivors in our study had a substantially higher prevalence of CH than age group‐matched healthy controls who were analyzed using the same assay. This potentially represents persistence of preleukemic clones as well as induction or selection of CH clones by cytotoxic chemotherapy.

The particularly high prevalence of CH variants in the *chemo* group is partly caused by the high frequency of *PPM1D* and *TP53* variants, which are known to be enriched in patients with prior exposure to cytotoxic chemotherapy. These variants were rarely found in survivors who had received an allogeneic transplantation, where the donor hematopoietic cells have not been exposed to cytotoxic chemotherapy. However, even *alloSCT* survivors exhibited a high prevalence of CH (54%). Tanaka et al. reported effective clearance of patient‐derived CH in AML patients in remission after alloSCT.^16^ Of note, the lower detection limit in the study by Tanaka et al. was 2.5% VAF, whereas the median VAF of variants we detected in *alloSCT* survivors was 1.2%, and in the absence of donor‐matched or longitudinal samples, we cannot rule out low‐level mixed chimerism and persistence of recipient‐derived CH clones in some survivors. However, previous studies suggest that most CH variants identified in transplant recipients are donor‐derived. A recent study utilizing ultra‐sensitive duplex sequencing on matched donor–recipient samples showed that CH variants are frequently transplanted from alloSCT donors to recipients, albeit not all of them were treated for AML.^26^

Prior studies of CH in alloSCT typically focused on donor samples and included patients with various underlying diagnoses. A study by Frick et al., including 50% AML patients, reported that CHIP mutations in alloSCT donors associated with an increased incidence of chronic Graft‐versus‐Host‐Disease and a higher risk of developing donor cell leukemia, while MDS or AML patients whose donors carried a CHIP mutation showed better OS.^29^ Similarly, in a cohort studied by Gibson et al. (35% AML survivors), *DNMT3A* CH mutations in alloSCT donors correlated with better OS and PFS in recipients.^30^ However, these groups did not systematically study CH in transplant recipients achieving long‐term survival.

Treatment modality did influence not only CH prevalence but also clone size and variant count. In *chemo* survivors, the prevalence of CH, clone size, and variant count increased with survivor age. CH in *chemo* survivors is of patient origin and likely represents a mixture of disease‐related (preleukemic) clones, unrelated age‐associated CH, and chemotherapy‐induced clones. On the other hand, most CH in survivors after allogeneic transplantation likely is donor‐derived. In *alloSCT* survivors, CH prevalence and variant count increased with time since transplantation. In a sub‐cohort of survivors with known alloSCT donor age, we were able to calculate the “hematopoietic age” of the stem/progenitor cell compartment and found it was a better predictor of CH status than time since transplantation alone. Donor type did not influence CH status. This finding aligns with data from Boettcher et al., who reported no association of clone size in alloSCT recipients with time since transplantation.^31^ Overall, despite the overall higher CH prevalence, CH dynamics in AML‐LTS show associations with stem cell age similar to those observed in the general population.

### Consequences of CH in AML survivors: associations with somatic disease

In persons without a history of AML, CH has been associated with an increased risk of developing numerous, often inflammation‐mediated diseases including coronary heart disease, heart failure, myocardial infarction, hypertension, ischemic stroke, type 2 diabetes, and cytopenias.^2^, ^12^, ^13^, ^32^, ^33^, ^34^, ^35^, ^36^, ^37^ CH has been also linked to smoking and obesity induced inflammation.^38^, ^39^ In our cohort of AML‐LTS, CH was not associated with hypertension, coronary heart disease, myocardial infarction, heart failure, or obesity, and we did not see any clinically relevant differences in blood counts according to CH status. However, AML survivors with CH ≥ 10% VAF had increased risks of diabetes. Further analysis revealed that diabetes was specifically associated with CH ≥ 10% VAF in *alloSCT* recipients, but not in *chemo* survivors. Although prior studies show an association between CH and incident diabetes in the general population, it is unclear whether this represents a causative relationship in our study.^13^ Our failure to identify other, previously reported associations of CH with somatic diseases in our AML‐LTS cohort might be a reflection of our limited sample size, as well as the survivorship bias inherent in our cross‐sectional study.

### Strengths and weaknesses of our study

To our knowledge, this is the first study investigating CH and its clinical implications in a large cohort of adult AML‐LTS. Strengths of our study include the long time interval after AML diagnosis, with a median of 11.6 y (range, 5.3–19.8 y), and our highly sensitive sequencing approach allowing us to detect variants down to 0.5% allelic frequency. This led to the detection of numerous low‐VAF variants, which would have been missed when using less sensitive sequencing techniques such as whole‐exome/genome sequencing or non‐error‐corrected targeted assays. Furthermore, the patients in our large, multicentric cohort were subjected to different treatment modalities and are representative of a wide range of AML survivors. However, due to its cross‐sectional design, our study is subject to survivorship bias, and severe post‐therapy events leading to death, including diseases potentially linked to CH such as stroke or myocardial infarction, would not be represented in our cohort. Due to our study design focusing on LTS, we may have missed high‐risk CH mutations, which might have induced somatic disease that led to death within 5 years from diagnosis. Thus, we might underestimate associations of CH with some somatic diseases in AML survivors. Furthermore, although our cohort is large for a cohort of AML‐LTS, it might be too small to detect weaker associations of CH with late and long‐term effects. Sample size and survivorship bias likely explain the missing association of CH with cardiovascular disease in our study. Furthermore, information on somatic health status was mostly collected through questionnaires from patients and their primary care providers and not ascertained by in‐person interviews or objective testing, which might have resulted in underreporting or misclassification of some endpoints. Despite these limitations, our study provides valuable insights into the spectrum and disease associations of CH in AML survivors. Future studies with larger, prospectively followed cohorts and structured clinical assessments will be necessary to clarify the full spectrum of CH‐associated risks, particularly in relation to diabetes, secondary neoplasms, and cardiovascular disease. Integrating genomic data with longitudinal clinical follow‐up could help shed more light on the clinical significance of CH and improve strategies for monitoring AML‐LTS.

## AUTHOR CONTRIBUTIONS

**Simon M. Krauß**: Conceptualization; data curation; methodology; software; investigation; formal analysis; visualization; writing—original draft; writing—review and editing. **Eva Telzerow**: Conceptualization; data curation; investigation; writing—review and editing. **Daniel Richter**: Conceptualization; formal analysis; investigation; methodology; writing—review and editing. **Anna S. Moret**: Data curation; investigation; writing—review and editing. **Maja Rothenberg‐Thurley**: Resources; writing—review and editing. **Cristina Sauerland**: Data curation; writing—review and editing. **Anne Weigert**: Writing—review and editing; resources. **Alessia Fraccaroli**: Writing—review and editing; resources. **Johanna Tischer**: Resources; writing—review and editing. **Frank Ziemann**: Writing—review and editing; resources. **Katharina S. Götze**: Writing—review and editing; resources. **Wolfgang E. Berdel**: Writing—review and editing; resources. **Bernhard Wörmann**: Writing—review and editing; resources. **Utz Krug**: Writing—review and editing; resources. **Jan Braess**: Writing—review and editing; resources. **Pia Heussner**: Writing—review and editing; resources. **Wolfgang Enard**: Writing—review and editing; funding acquisition; supervision; conceptualization. **Wolfgang Hiddemann**: Writing—review and editing; resources. **Karsten Spiekermann**: Writing—review and editing; resources. **Dennis Görlich**: Conceptualization; data curation; formal analysis; writing—review and editing. **Uwe Platzbecker**: Writing—review and editing; supervision; funding acquisition. **Klaus H. Metzeler**: Writing—review and editing; writing—original draft; conceptualization; funding acquisition; supervision.

## CONFLICT OF INTEREST STATEMENT

The authors declare no conflicts of interest.

## ETHICS STATEMENT

This study was approved by the responsible ethics committee at the University of Munich (reference no. 17‐444). All patients provided written informed consent to participate in this study. This study was registered in the German Clinical Trials Registry (DRKS, www.drks.de, identifier DRKS00023991).

## FUNDING

This work was funded by the German José Carreras Foundation (DJCLS, project 13R/2019) and the Deutsche Forschungsgemeinschaft (DFG, German Research Foundation)—SFB 1243 “Cancer evolution.”

## Supporting information

Supporting Information.

Supporting Information.

## Data Availability

The data that support the findings of this study are available on request from the corresponding author. The data are not publicly available due to privacy or ethical restrictions.
