# Supplementary material for: Clonal hematopoiesis in AML long‐term survivors: Risk factors and clinical consequences
Source: Hemasphere. 2025 Jul 24;9(7):e70183. doi: 10.1002/hem3.70183 (PMC12288099; doi:10.1002/hem3.70183)
Supplement: Supplementary file 1 — Supporting Information. [file HEM3-9-e70183-s001.docx]

Supplemental Appendix

Clonal Hematopoiesis in AML Long-Term Survivors:
Risk Factors and Clinical Consequences

# Supplemental methods

## Single molecule molecular inversion probe (smMIP) assay design

Single molecule molecular inversion probes were designed using MIPgen^1^ as described previously^2^, with the following changes: The original linker sequence (“NNNNNCTTCAGCTTCCCGATATCCGACGGTAGTGTNNNNN”) was replaced by a custom design (“NNNNNAGATCGGAAGAGCGTGTGTATAAGAGACAGNNNNN”) to facilitate sequencing on Illumina sequencers without the addition of custom sequencing primers. The probe panel was manually curated to achieve “alternating single coverage”, i.e. each genomic position is covered either on the sense or antisense DNA strand, with neighboring smMIPs covering opposite strands whenever possible, to reduce steric hindrance during hybridization to the template DNA. The smMIPs were purchased from Sigma-Aldrich (Merck) at 25 nmol scale normalized to a concentration of 50 µM. A full list of smMIPs can be found in table S2.

## Sequencing library preparation

The smMIP assay setup follows a previously published protocol^2,3^ with minor modifications. Individual smMIPs were pooled equimolarly and phosphorylated as described previously. After initial denaturation of DNA at 95° C for 10 min the hybridization capture was performed for 18 h at 60 °C in 25 µl reactions with 100 ng of template DNA. The reaction mixture contained 5 µL of phosphorylated smMIP pool resulting in a ratio of 1000 smMIP molecules per haploid genome. Next, each reaction was treated by exonuclease digestion and two technical replicates of 12.5 µL were added to 50 µL PCR reactions for amplification and barcoding. Technical PCR replicates were pooled, purified twice via a custom-made mixture of paramagnetic beads containing SpeedBeads (Sigma-Aldrich) at 0.8x, verified via agarose gel electrophoresis, and quantified via QuantIt Picogreen (Invitrogen, Thermo Fisher Scientific), followed by equimolar pooling of all libraries and quality control of the library pool using a BioAnalyzer (Agilent). Sequencing was performed on an Illumina HiSeq 1500 platform with 2x 100 bp paired-end sequencing to a median depth of 4.7 million read pairs per library. To optimize its performance, the original smMIP pool was tested on control DNA, and the sequencing coverage of each probe was evaluated to identify underperforming smMIPs. The relative concentration of these probes in the pool was increased to either 10x, 25x or 50x according to their performance. Each sample was prepared in two technical replicates.

## Data processing and variant calling

Raw sequencing data were demultiplexed using deML^4^ (ver. 1.1.3), followed by quality control using fastQC. UMIs were moved to the read header via fastp^5^ (ver. 0.21.0) and extended by two replicate-specific bases, to be able to differentiate UMIs from different replicates. Afterwards, sequencing reads from replicates were merged. The smMIP hybridization arms were removed via cutadapt^6^ (ver. 3.4), and reads were mapped to the human genome (hg19) using bwa mem^7^ (ver. 0.7.17-r1188). Tools from GATK4^8^ (ver. 4.2.1.0) were used for pre-processing of the mapped data (AddOrReplaceReadGroups, UmiAwareMarkDuplicatesWithMateCigar, BaseRecalibrator, ApplyBQSR) and for variant calling (Mutect2, FilterMutectCalls). Bcftools^9^ (ver. 1.8) was employed for splitting of multiallelic rows and left-normalization of indels. Annotation of variants was performed via ANNOvar^10^.

## Error profile generation

The mapped reads were processed using Gencore^11^ (ver. 0.16) to create uniquely mapped read files. Pileups of these files were created using samtools^9^ (ver. 1.13), and coverage as well as non-reference bases (nucleotide changes, insertions, deletions) were counted at each position covered by the smMIP panel for each sample to calculate the per-sample error rate. From the per-sample error rates we calculated the average error rate for each covered position across the whole cohort. Of note, whenever the error rate for a position in a sample exceeded 5%, we deemed this to be a true variant and thus excluded this sample from the error profile generation for this specific position.

## Variant filtering

All variants were filtered in R^12^ (ver. 3.6.0) using functions from tidyverse^13^ (ver. 1.3.1) packages by the following criteria: must pass Mutect2 filters; >= 5 unique reads confirming the variant (except when annotated as ”haematopoietic” in Cosmic (ver. 91), then >= 3 unique reads); variant must be exonic or splicing related and must not be synonymous; VAF must be >= 0.5%; probability of being background error must be <= 0.005 (Poisson test; corrected for multiple testing via Benjamini-Hochberg false discovery rate correction); variant VAF must be < 80%; variant VAF must be < 40% if frequency in gnomAD^14^ is >= 0.1% or in ExAC^14^ >= 1% and variant is not annotated as “myeloid” in ClinVar^15^. Variants were furthermore filtered if they occurred in known artifact-prone regions of genes or our panel and then once more by manual curation via IGV^16^.

# Supplemental references

1. Boyle EA, O’Roak BJ, Martin BK, Kumar A, Shendure J. MIPgen: optimized modeling and design of molecular inversion probes for targeted resequencing. *Bioinformatics*. 2014;30(18):2670-2672. doi:10/f6j65d

2. Acuna-Hidalgo R, Sengul H, Steehouwer M, et al. Ultra-sensitive Sequencing Identifies High Prevalence of Clonal Hematopoiesis-Associated Mutations throughout Adult Life. *Am J Hum Genet*. 2017;101(1):50-64. doi:10.1016/j.ajhg.2017.05.013

3. Hiatt JB, Pritchard CC, Salipante SJ, O’Roak BJ, Shendure J. Single molecule molecular inversion probes for targeted, high-accuracy detection of low-frequency variation. *Genome Res*. 2013;23(5):843-854. doi:10/f4v9nz

4. Renaud G, Stenzel U, Maricic T, Wiebe V, Kelso J. deML: robust demultiplexing of Illumina sequences using a likelihood-based approach. *Bioinformatics*. 2015;31(5):770-772. doi:10.1093/bioinformatics/btu719

5. Chen S, Zhou Y, Chen Y, Gu J. fastp: an ultra-fast all-in-one FASTQ preprocessor. *Bioinformatics*. 2018;34(17):i884-i890. doi:10.1093/bioinformatics/bty560

6. Martin M. Cutadapt removes adapter sequences from high-throughput sequencing reads. *EMBnet.journal*. 2011;17(1):10-12. doi:10.14806/ej.17.1.200

7. Li H, Durbin R. Fast and accurate short read alignment with Burrows–Wheeler transform. *Bioinformatics*. 2009;25(14):1754-1760. doi:10.1093/bioinformatics/btp324

8. Benjamin D, Sato T, Cibulskis K, Getz G, Stewart C, Lichtenstein L. Calling Somatic SNVs and Indels with Mutect2. Published online December 2, 2019. doi:10.1101/861054

9. Danecek P, Bonfield JK, Liddle J, et al. Twelve years of SAMtools and BCFtools. *GigaScience*. 2021;10(2):giab008. doi:10.1093/gigascience/giab008

10. Wang K, Li M, Hakonarson H. ANNOVAR: functional annotation of genetic variants from high-throughput sequencing data. *Nucleic Acids Research*. 2010;38(16):e164. doi:10.1093/nar/gkq603

11. Chen S, Zhou Y, Chen Y, et al. Gencore: an efficient tool to generate consensus reads for error suppressing and duplicate removing of NGS data. *BMC Bioinformatics*. 2019;20(23):606. doi:10.1186/s12859-019-3280-9

12. R Core Team. R: A Language and Environment for Statistical Computing. Published online 2019. https://www.R-project.org/

13. Wickham H, Averick M, Bryan J, et al. Welcome to the tidyverse. *Journal of Open Source Software*. 2019;4(43):1686. doi:10.21105/joss.01686

14. Karczewski KJ, Francioli LC, Tiao G, et al. The mutational constraint spectrum quantified from variation in 141,456 humans. *Nature*. 2020;581(7809):434-443. doi:10.1038/s41586-020-2308-7

15. Landrum MJ, Lee JM, Riley GR, et al. ClinVar: public archive of relationships among sequence variation and human phenotype. *Nucleic Acids Research*. 2014;42(D1):D980-D985. doi:10.1093/nar/gkt1113

16. Robinson JT, Thorvaldsdóttir H, Winckler W, et al. Integrative Genomics Viewer. *Nat Biotechnol*. 2011;29(1):24-26. doi:10.1038/nbt.1754

# Supplemental figures

## Figure S1


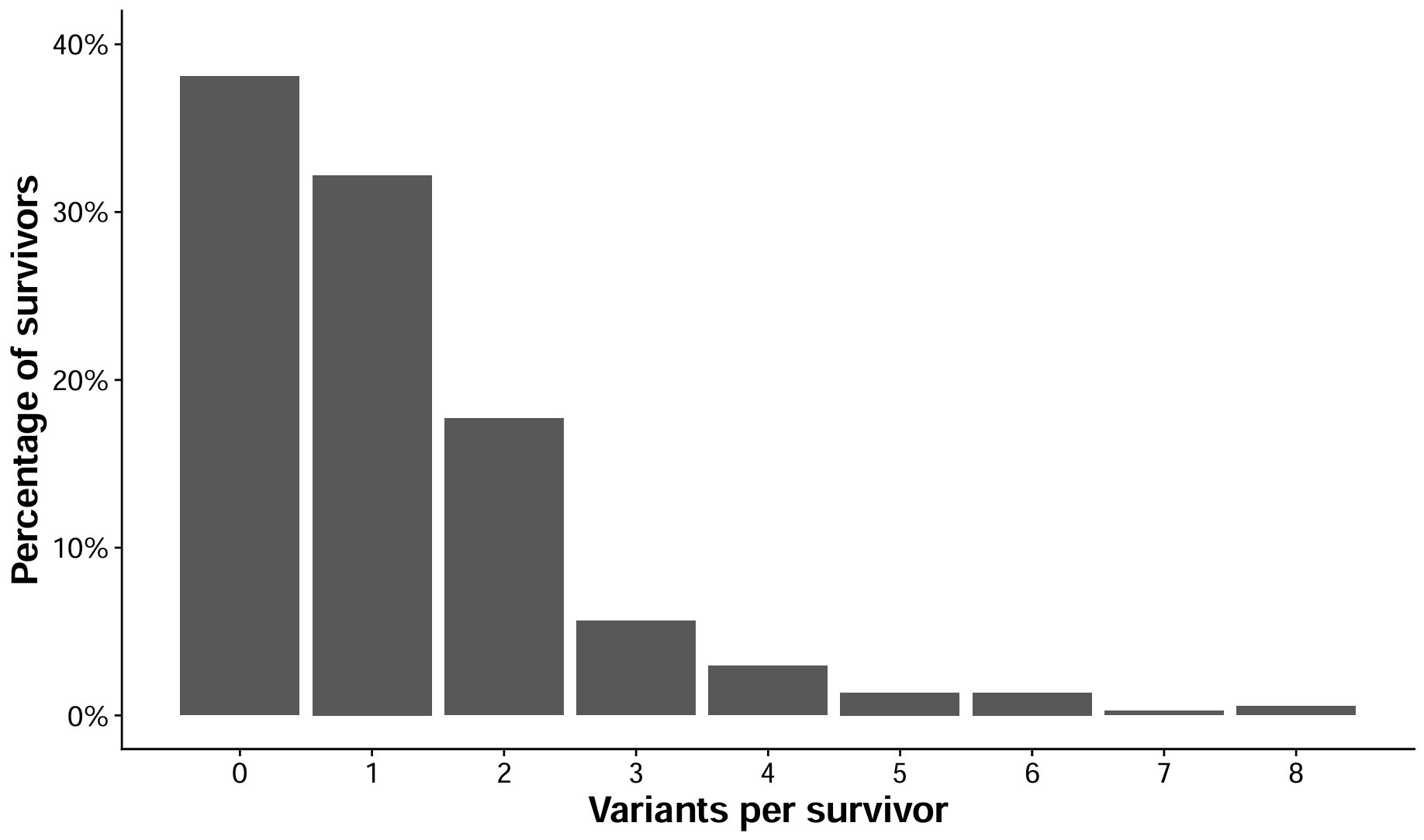


**Figure S1:** Distribution of variant count per survivor displayed as bar plot.

## Figure S2


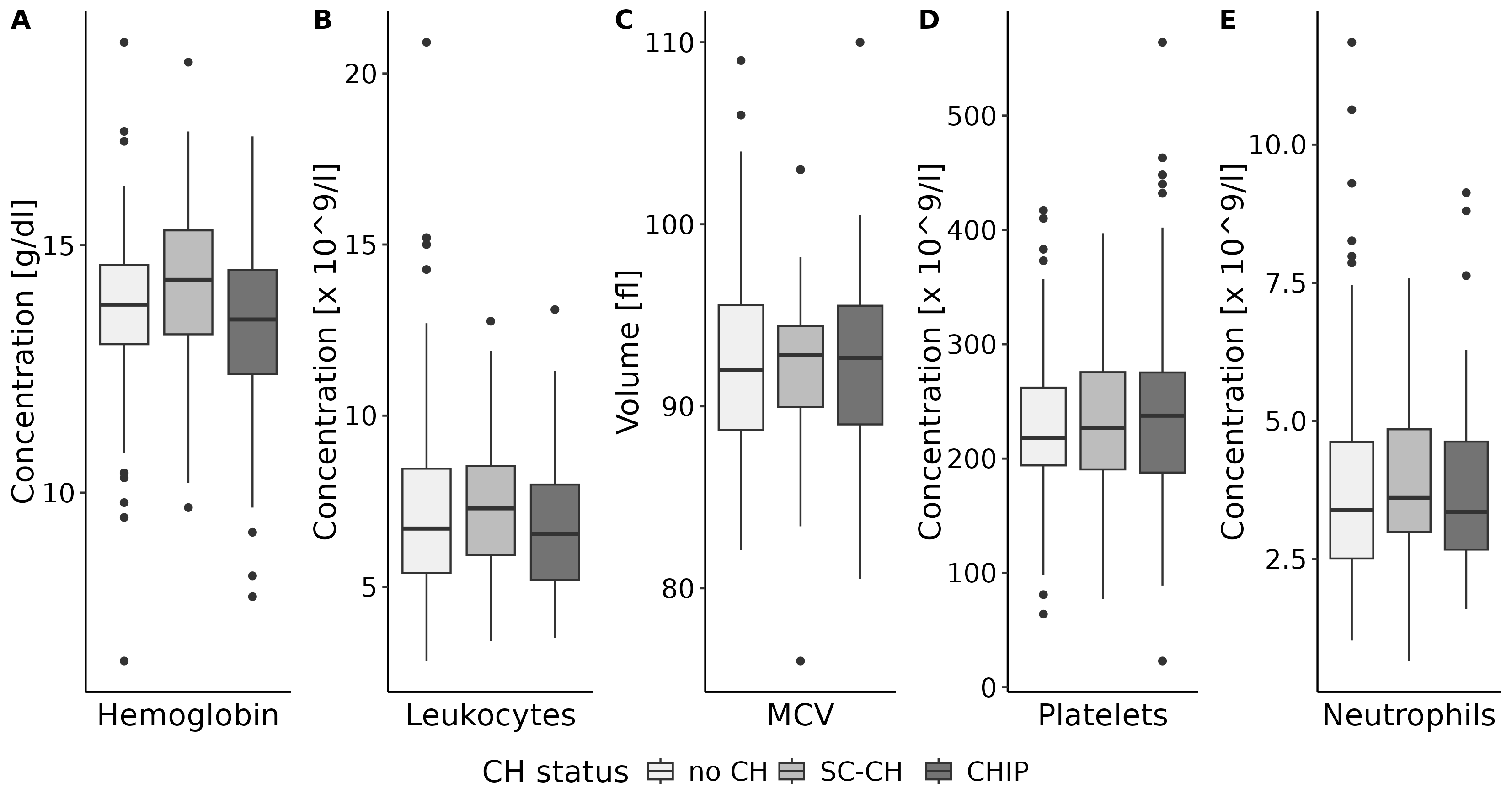


**Figure S2:** Overview about blood count data stratified by CH status displayed as boxplots for all survivors with available data. **A)** Hemoglobin concentration. **B)** Leukocyte concentration. **C)** Mean corpuscular volume (MCV). **D)** Platelet concentration. **E)** Neutrophil concentration.

## Figure S3


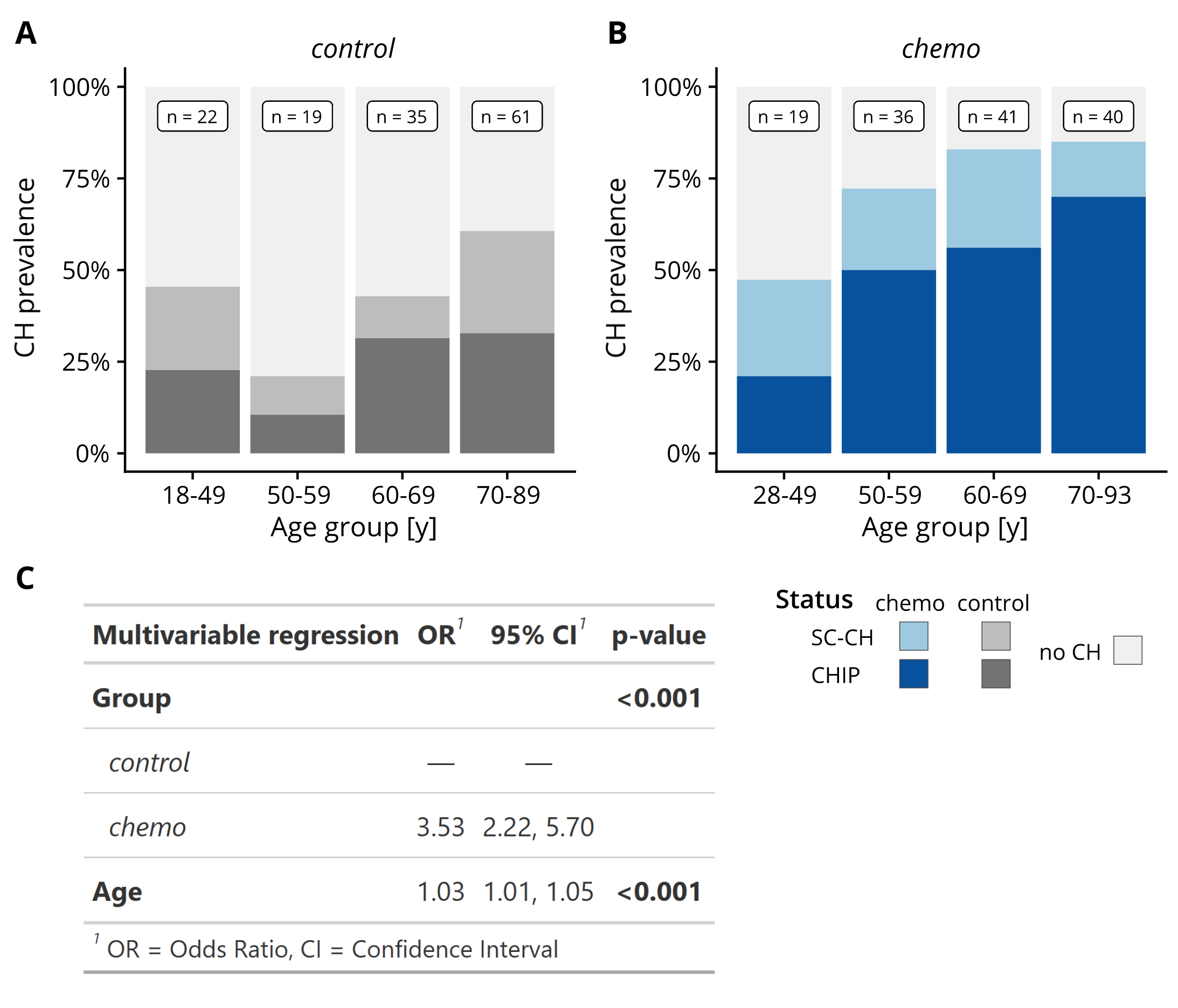


**Figure S3**: Comparison of CH prevalence and age dependence in control cohort and *chemo* survivors. **A+B)** Stacked barplots displaying SC-CH and LC-CH prevalence in each age group. **C)** Results of multivariable ordinal logistic regression modelling CH status in dependence of group (control cohort or *chemo* survivor) and age.

## Figure S4


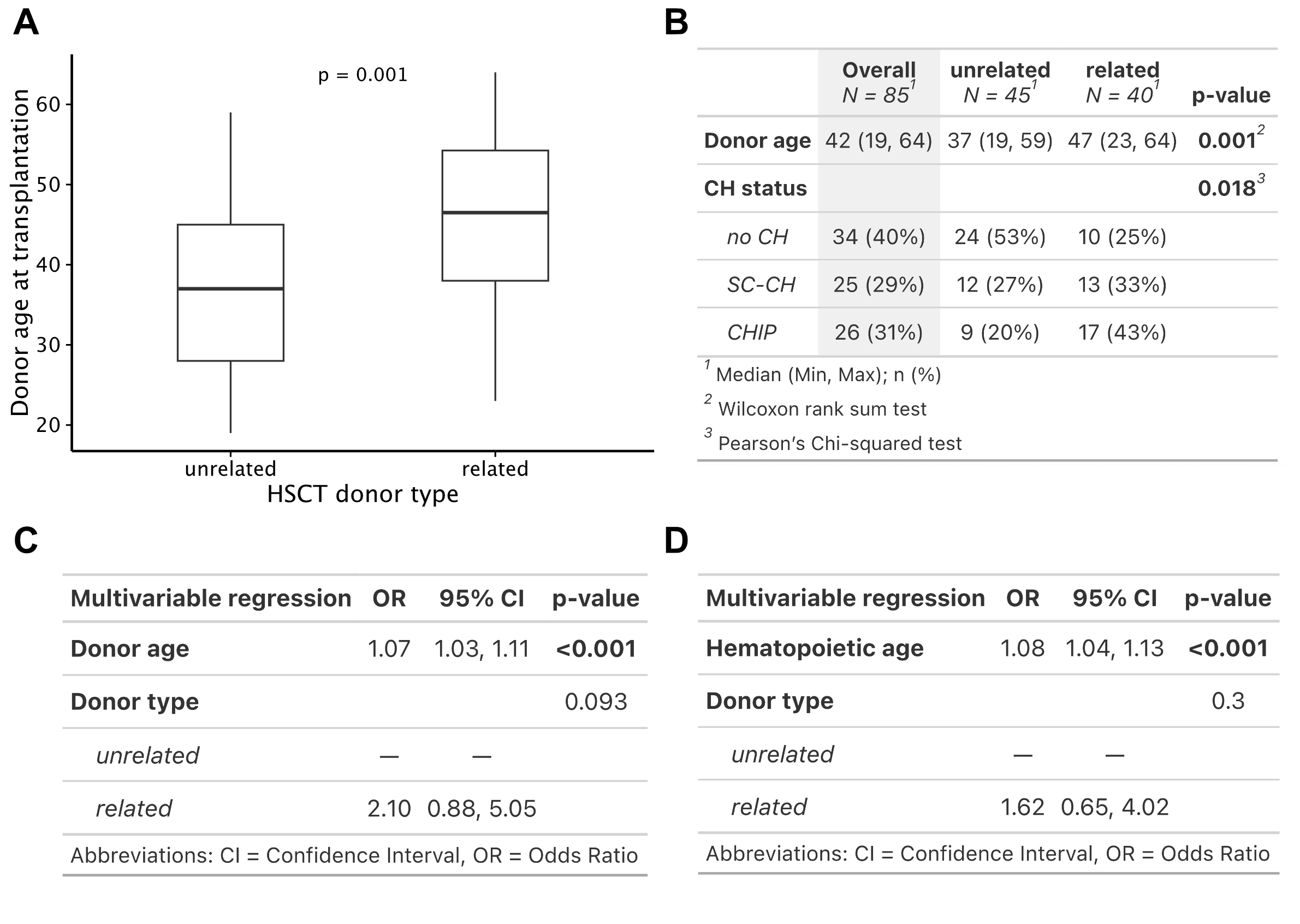


**Figure S4**: Associations between donor type, donor age and survivor CH status. **A+B**) Boxplot and table displaying donor age by relation status (unrelated vs. related), with (**B**) additionally displaying CH status of survivor by donor type. **C+D**) Multivariable ordinal logistic regression models showing the influence of donor age (**C**) or hematopoietic age (**D**) on CH prevalence, adjusted by donor type. Age was treated as a continuous variable and odds ratio (OR) is shown per year.

## Figure S5


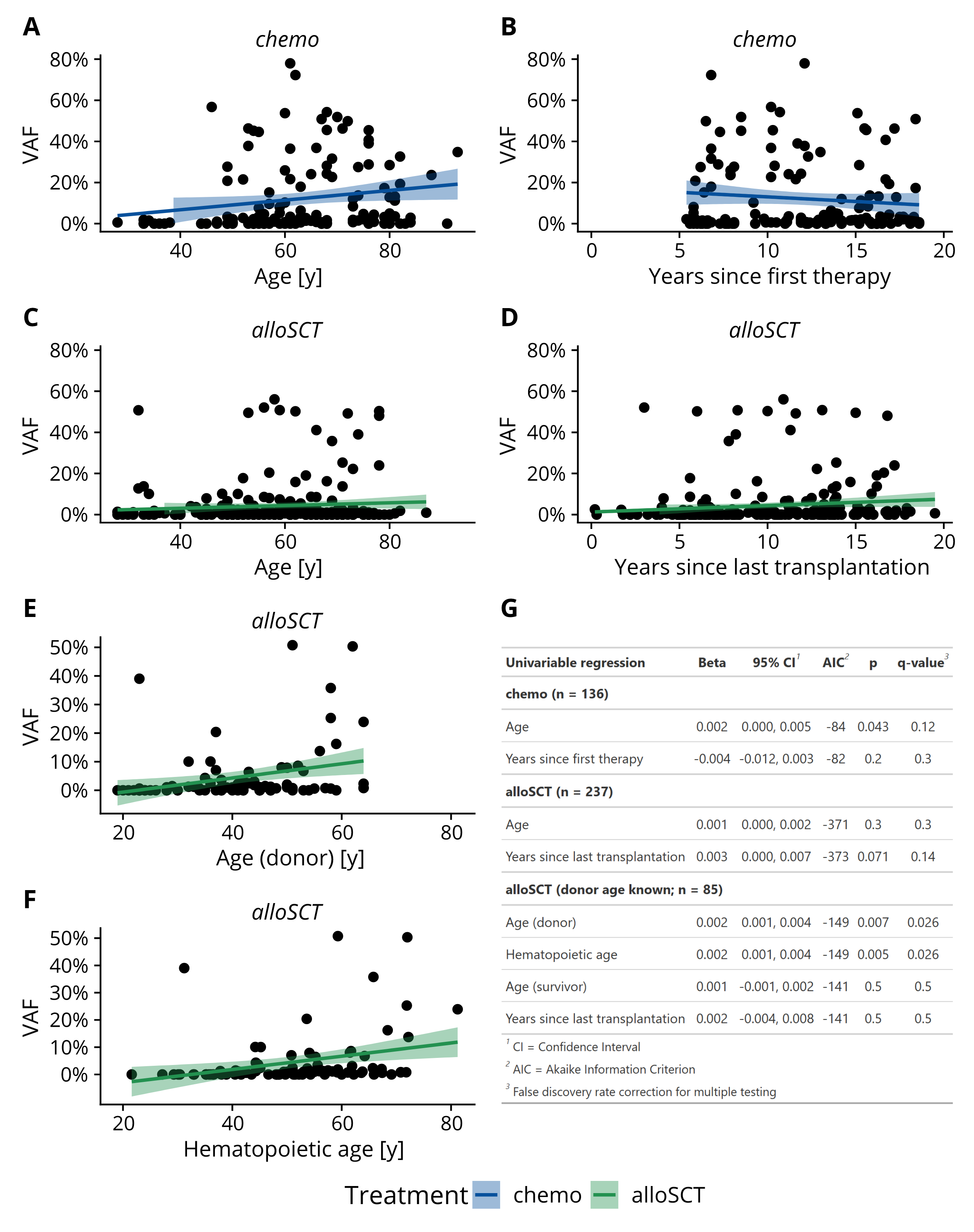


**Figure S5:** Associations between time factors and variant allele frequency (VAF) of the largest variant per survivor (including 0% VAF for survivors without mutations) displayed as scatterplot with overlayed linear regression curves (**A-F**) or as table with the results of univariate linear regression models (**G**). For simplicity, we only considered the largest variant per survivor as to avoid modelling dependencies or correlations of multiple variants in the same survivor.

## Figure S6


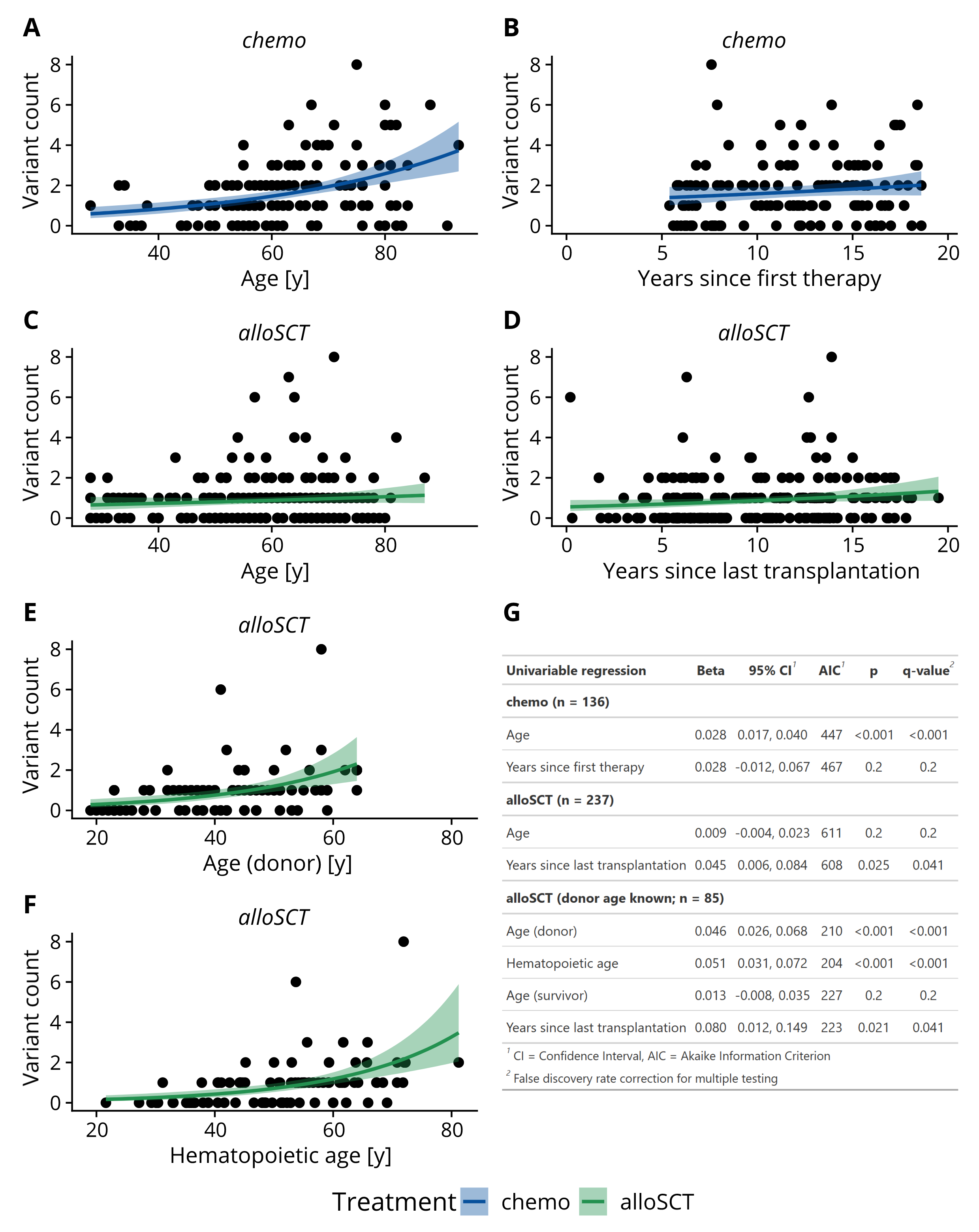


**Figure S6:** Associations between time factors and variant count in survivors displayed as scatterplot with overlayed regression curves (**A-F**) or as table with the results of univariable regression models (**G**). Univariable negative binomial regression was chosen due to overdispersion of data.

## Figure S7


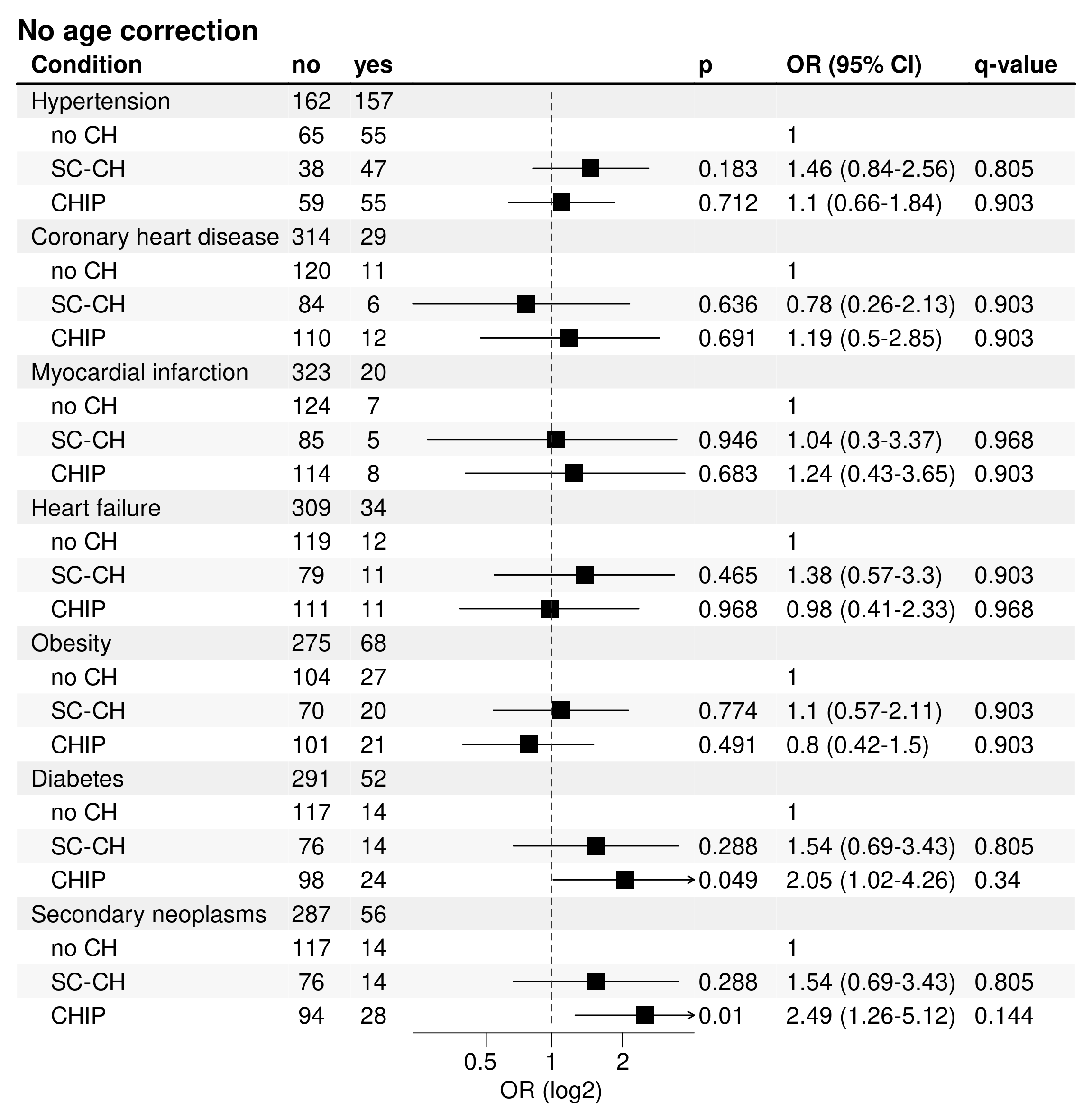


**Figure S7:** Association between CH and somatic comorbidities presented as forest plot. OR, CI and p-values were calculated via univariable logistic regression. No/yes columns display number of survivors affected by each comorbidity.

# Supplemental tables

## Table S1: List of smMIP target loci

See supplementary excel file.

## Table S2: smMIP list

See supplementary excel file.

## Table S3: List of all detected CH variants

See supplementary excel file.

## Table S4: Overview of treatment schemata and number of lines of chemotherapy.

| **Variable** | **Overall** | **Chemo-therapy only** | **alloSCT** | **p** | **q-value** |
| --- | --- | --- | --- | --- | --- |
| Survivor number | N = 373 | n = 136 | n = 237 |  |  |
| **Number of induction + consolidation cycles (mean, range)** | 2.7 (1, 5) | 2.9 (1, 5) | 2.6 (1, 5) | **< 0.001^1^** | < 0.001 |
| **Induction therapy regimen (n, %)** |  |  |  |  |  |
| *7+3* | 26 (7.0%) | 4 (2.9%) | 22 (9.3%) |  |  |
| *7+3 - FLAG-Ida* | 4 (1.1%) | 0 (0%) | 4 (1.7%) |  |  |
| *7+3 - HAM* | 1 (0.3%) | 0 (0%) | 1 (0.4%) |  |  |
| *FLAG-Ida* | 1 (0.3%) | 0 (0%) | 1 (0.4%) |  |  |
| *FLAMSA* | 2 (0.5%) | 0 (0%) | 2 (0.8%) |  |  |
| *HAM* | 24 (6.4%) | 10 (7.4%) | 14 (5.9%) |  |  |
| *HAM-HAM* | 82 (22%) | 27 (20%) | 55 (23%) |  |  |
| *TAD* | 16 (4.3%) | 10 (7.4%) | 6 (2.5%) |  |  |
| *TAD-HAM* | 110 (29%) | 51 (38%) | 59 (25%) |  |  |
| *sHAM* | 106 (28%) | 34 (25%) | 72 (30%) |  |  |
| *unknown* | 1 (0.3%) | 0 (0%) | 1 (0.4%) |  |  |
| **Number of induction therapy cycles (mean, range)** | 1.9 (1, 2) | 1.8 (1, 2) | 1.9 (1, 2) | 0.3^1^ | 0.3 |
| **Consolidation therapy regimen (n, %)** |  |  |  |  |  |
| *Autologous PBSCT* | 22 (5.9%) | 18 (13%) | 4 (1.7%) |  |  |
| *HAM* | 1 (0.3%) | 0 (0%) | 1 (0.4%) |  |  |
| *HiDAC* | 20 (5.4%) | 6 (4.4%) | 14 (5.9%) |  |  |
| *TAD/HiDAC* | 3 (0.8%) | 0 (0%) | 3 (1.3%) |  |  |
| *TAD* | 216 (58%) | 101 (74%) | 115 (49%) |  |  |
| *none* | 110 (29%) | 10 (7.4%) | 100 (42%) |  |  |
| *unknown* | 1 (0.3%) | 1 (0.7%) | 0 (0%) |  |  |
| **Number of consolidation therapy cycles (mean, range)** | 0.8 (0, 4) | 1.0 (0, 3) | 0.7 (0, 4) | **<0.001^1^** | <0.001 |
| **Maintenance therapy regimen (n, %)** |  |  |  |  |  |
| *AMLCG-type maintenance* | 134 (36%) | 84 (62%) | 50 (21%) |  |  |
| *AMLCG-type maintenance + DC vaccination* | 2 (0.5%) | 2 (1.5%) | 0 (0%) |  |  |
| *Histamine dihydrochloride + IL-2* | 3 (0.8%) | 2 (1.5%) | 1 (0.4%) |  |  |
| *none* | 234 (63%) | 48 (35%) | 186 (78%) |  |  |
| **Number of maintenance therapy cycles (mean, range)** | 12.3 (1, 38) | 15.3 (1, 38) | 7.1 (1, 36) | **<0.001**^1^ | <0.001 |

**Notes:** Treatment regimens for AMLCG trials are described in the following publications: AMLCG-1999: Krug U et al., Leukemia 30, 1230–1236; AMLCG-2004: Braess J et al., *Blood*. 2009;113(17):3903-3910 and AMLCG-2008: Braess, J. et al. Leukemia 32, 2558–2571.

**Abbreviations:** 7+3: cytarabine (7 days) and daunorubicin (3 days); FLAG-Ida: Fludarabine, Cytarabine, Idarubicin, and G-CSF, FLAMSA: Fludarabine, Amsacrine, Cytarabine; HAM: High-dose cytarabine and mitoxantrone; sHAM: sequential (dose-dense) HAM; TAD: Thioguanine, cytarabine, daunorubicin, HiDAC: high-dose cytarabine.
^1^ Wilcoxon rank sum test

## Table S5: Therapy lines in survivors who had not undergone alloSCT by CH status

| **Variable** | **Overall** | **no CH** | **SC-CH** | **CHIP** | **p** | **q-value** |
| --- | --- | --- | --- | --- | --- | --- |
| Survivor number | N = 136 | n = 33 | n = 30 | n = 73 |  |  |
| **Intensive therapy cycles** **(Induction+Consolidation) (mean, range)** | 2.9 (1, 5) | 2.9 (2, 5) | 2.9 (2, 5) | 2.8 (1, 5) | 0.7^1^ | >0.9 |
| *unknown* | 2 | 1 | 0 | 1 |  |  |
| **Induction cycles (mean, range)** | 1.8 (1, 2) | 1.9 (1, 2) | 1.9 (1, 2) | 1.8 (1, 2) | 0.4^1^ | 0.8 |
| **Consolidation cycles (mean, range)** | 1.0 (0, 3) | 1.0 (0, 3) | 1.0 (0, 3) | 1.0 (0, 3) | >0.9^1^ | >0.9 |
| *unknown* | 2 | 1 | 0 | 1 |  |  |
| **Maintenance cycles**  **(mean, range)** | 15.3  (1, 38) | 16.6  (1, 38) | 11.6  (1, 29) | 15.9  (1, 38) | 0.3^1^ | 0.8 |
| *unknown* | 48 | 10 | 13 | 25 |  |  |

^1^ Kruskal-Wallis rank sum test

## Table S6: Matching status of allogeneic hematopoietic stem cell donors by CH status.

| **Variable** | **Overall** | **no CH** | **SC-CH** | **CHIP** | **p** |
| --- | --- | --- | --- | --- | --- |
| Survivor number | N = 237 | n = 109 | n = 67 | n = 61 |  |
| **HSCT donor** |  |  |  |  | 0.2 |
| *MRD* | 73 (32%) | 25 (25%) | 21 (33%) | 27 (45%) |  |
| *HAPLO* | 7 (3.1%) | 5 (4.9%) | 1 (1.6%) | 1 (1.7%) |  |
| *MUD* | 118 (52%) | 60 (59%) | 33 (52%) | 25 (42%) |  |
| *mmMUD* | 27 (12%) | 12 (12%) | 8 (13%) | 7 (12%) |  |
| *unknown* | 12 | 7 | 4 | 1 |  |

**Abbreviations:** MRD: matched-related donor, Haplo: haploidentical donor, MUD: matched-unrelated donor, mmMUD: mismatched-unrelated donor

## Table S7: Clinical and demographic characteristics of AML long-term survivors stratified by treatment modality.

| **Variable** | **Overall** | **chemo** | **alloSCT** | **p** |
| --- | --- | --- | --- | --- |
| Survivor number | N = 373^1^ | n = 136^1^ | n = 237^1^ |  |
| **Age at initial diagnosis [y]** | 49 (17, 80) | 51 (18, 80) | 49 (17, 74) | 0.067² |
| **Age at CH analysis [y]** | 61 (28, 93) | 62 (28, 93) | 59 (28, 87) | **0.007**² |
| **Years since AML diagnosis** | 11.6  (5.3, 19.8) | 12.4  (5.5, 18.6) | 10.8  (5.3, 19.8) | **0.003**² |
| **Sex** |  |  |  | 0.14³ |
| *female* | 216 | 72 (33%) | 144 (67%) |  |
| *male* | 157 | 64 (41%) | 93 (59%) |  |
| **Smoker** | 61 | 23 (38%) | 38 (62%) | 0.9³ |
| *unknown* | 61 | 21 | 40 |  |
| **AML type** |  |  |  | **<0.001**³ |
| *de novo AML* | 313 | 126 (40%) | 187 (60%) |  |
| *sAML/tAML* | 60 | 10 (17%) | 50 (83%) |  |
| **Cytogenetic risk group** |  |  |  | **<0.001**³ |
| *favorable* | 51 | 34 (67%) | 17 (33%) |  |
| *intermediate* | 266 | 99 (37%) | 167 (63%) |  |
| *adverse* | 56 | 3 (5.4%) | 53 (95%) |  |
| **Leukocyte count at**  **initial diagnosis (x 10^9^/l)** | 10.2  (0.6, 391.2) | 14.8  (0.9, 391.2) | 8.1  (0.6, 284.0) | 0.067² |
| *unknown* | 12 | 5 | 7 |  |
| **CH status** |  |  |  | **<0.001**³ |
| *no CH* | 142 | 33 (23%) | 109 (77%) |  |
| *SC-CH* | 97 | 30 (31%) | 67 (69%) |  |
| *CHIP* | 134 | 73 (54%) | 61 (46%) |  |
| **AMLCG Trial** |  |  |  | **0.016**³ |
| *1999* | 215 | 93 (43%) | 122 (57%) |  |
| *2004* | 24 | 7 (29%) | 17 (71%) |  |
| *2008* | 67 | 19 (28%) | 48 (72%) |  |
| *Registry* | 67 | 17 (25%) | 50 (75%) |  |

^1^ Median (range); n (row %)
^2^ Wilcoxon rank sum test
^3^ Pearson’s Chi-squared test

## Table S8: Secondary neoplasms detected in AML long-term survivors

| **Type of neoplasm** | **Number of incident cancers (alloSCT patients)** | **Number of incident cancers (chemo patients)** |
| --- | --- | --- |
| non-melanoma skin cancer | 12 | 4 |
| GI cancer | 4 | 3 |
| head and neck cancer | 4 | 0 |
| other | 4 | 1 |
| CNS tumor | 3 | 1 |
| prostate cancer | 3 | 1 |
| bladder cancer | 2 | 1 |
| lung cancer | 2 | 1 |
| breast cancer | 1 | 5 |
| lymphoid malignancy | 1 | 1 |
| melanoma | 1 | 1 |

**Note:** “Other” neoplasms included vulvar intraepithelial neoplasia, thyroid carcinoma, and ovarian carcinoma in the *alloSCT* subgroup and liposarcoma in the *chemo* subgroup.
